# Editorial: Emerging anti-cancer compounds and immunomodulators for pancreatic cancer treatment

**DOI:** 10.3389/fonc.2024.1411836

**Published:** 2024-04-19

**Authors:** Kathleen E. DelGiorno, Stephen Safe, Jennifer M. Bailey-Lundberg

**Affiliations:** ^1^ Department of Cell and Developmental Biology, Vanderbilt University, Nashville, TN, United States; ^2^ Department of Veterinary Physiology and Pharmacology, Texas A&M University, College Station, TX, United States; ^3^ Department of Anesthesiology, Critical Care, and Pain Medicine, McGovern Medical School, The University of Texas Health Science Center at Houston, Houston, TX, United States

**Keywords:** pancreatic adenocarcinoma, immunotherapy combined therapy, artificial intelligence, therapeutics, chemotherapy - oncology

Several recent studies have shown that cancers arising in the gastrointestinal tract are on the rise, especially in younger adults. Of those gastrointestinal cancers, pancreatic adenocarcinoma, a subtype of pancreatic cancer, remains one of the deadliest (1). Pancreatic adenocarcinoma is expected to surpass colorectal cancer-related deaths by the year 2030 to become the second leading cause of cancer-related deaths in the United States (2). Despite advances in radiation therapy, immune-oncology, surgery, and new therapeutics, the 5-year survival rate for all stages is 12%. Several factors contribute to the poor prognosis for these patients including a hostile, hypoxic and immune suppressed tumor microenvironment, limited approaches for early detection, minimal surgical resection options for most patients who are diagnosed with locally advanced or metastatic pancreatic cancer, and other complications including malignant ascites, as reviewed by Han and Borazanci. In a systematic review published by Su et al., a significant association between early incidence of venous thromboembolism and poorer overall survival in patients with pancreatic cancer indicates another clinical consideration in understanding overall survival rates for this malignancy.

There are several emerging therapeutic strategies for treating pancreatic cancer. In a review by Tindall et al., therapeutic strategies targeting the TGF-β family are considered with an emphasis on the stage of disease. Targeting TGF-β has gained traction for pancreatic cancer as pathway activation can promote immune suppression and extracellular matrix production, two critical components of the pancreatic cancer microenvironment that inhibit the function of chemo and immunotherapeutic agents. Studies by Wang et al. have discovered a new agent called C150 that inhibits epithelial to mesenchymal transition (EMT) through enhancement of proteosome assembly and subsequent degradation of transcription factors important for epithelial to mesenchymal transition. Experiments were conducted in an orthotopic model of pancreatic cancer and treatment with C150 (150 mg/kg 3x weekly) significantly increased survival of mice showing strong preclinical consideration.

IL-6 overexpression has been associated with poor prognosis in patients with pancreatic cancer. Leukemia inhibitory factor (LIF) is a cytokine that belongs to the IL-6 family. LIF mediates intracellular signaling by binding to a heterodimeric receptor complex including LIF receptor and Gp130. A recent study by Di Giorgo et al. showed BAR502, a non-bile acid steroidal ligand for two LIF receptors, Farnesoid-X-Receptor (FXR) and G Protein Bile Acid Activated Receptor (GPBAR1), reduced binding of LIF to the LIF receptor complex and reduced proliferation of MIA PaCa-2 pancreatic cancer cells.

An emerging consideration for therapeutic targeting is Claudin18.2, a tight junction protein highly expressed in pancreatic cancer primary tumors and in metastatic lesions. There are several clinical trials targeting Claudin18.2, as reviewed in Xu et al., and a number of emerging strategies to target Claudin18.2 including monoclonal antibodies, antibody-drug conjugates, bispecific antibodies, and a CAR-T cell drug targeting Claudin18.2, also currently being evaluated in clinical trials.

Development of more effective treatment of metastatic pancreatic cancer is critically needed for patients diagnosed with unresectable pancreatic adenocarcinoma. In a recent study published by Lu et al., third-line treatment for patients with metastatic pancreatic cancer prolonged the survival time of patients. In this study, survival was evaluated in 72 patients, 36 of whom received chemotherapy alone, 16 who received chemotherapy combined with targeted therapy or immunotherapy, 14 who received chemotherapy-free anti-tumor agents, and 6 who received palliative care. While the data show improved survival with chemotherapy, the study also revealed that third-line treatment with targeted therapy or immunotherapy did not improve survival benefits to chemotherapy alone and was associated with more adverse side effects. In a somewhat related study published by Cheng et al., patients who were diagnosed with stage III/IV pancreatic cancer were assigned into groups based on treatment with programmed cell death protein 1 (PD-1) blockade plus gemcitabine and nab-paclitaxel or chemotherapy alone. The patients treated with PD-1/chemotherapy had a progression free survival of 8 months as compared to 3.5 months in the chemotherapy alone cohort and the median overall survival was 15 months in the PD-1/chemotherapy arm as compared to 8 months in the chemotherapy alone arm. This study is timely as immunotherapeutic strategies targeting PD-1 in combination with other strategies have not previously shown survival comparison in patients with pancreatic cancer.

Future clinical trials will need to evaluate overall response to therapy to assist in treating this aggressive gastrointestinal malignancy. Additionally, expanded efforts in early detection are promising to aid in the diagnosis of patients with resectable early-stage cancer, who qualify for surgical resection, which has a more promising outlook for survival. Artificial Intelligence (AI) uses machines to reproduce human cognition and learning. AI methods are under evaluation for assisting with early screening, diagnosis, surgical treatment, risk prediction and management of post operative complications for patients with pancreatic cancer (reviewed in Zhao et al.). In the field of early detection and the diagnosis of intraductal papillary mucinous neoplasia or pancreatic adenocarcinoma, deep learning models have emerged with superior performance and high diagnostic accuracy. Additionally, in this review, the use of deep learning models and algorithms enabled risk prediction models for postoperative complications with strong area under the curve measures, indicating AI through the amalgamation of imaging modalities, tree models and AI-driven random forest and neural network algorithms can aid in the postoperative care of patients. Combined use of AI, immune-oncology and radiation, ablation, and new therapeutic approaches are all promising for the future care and management of pancreatic cancer.

## Author contributions

KD: Writing – review & editing. SS: Writing – review & editing. JB-L: Writing – original draft.
